# Drug interactions between cephalosporins and 5-FU-based chemotherapy in the treatment of patients with gastrointestinal cancer - an exploratory cohort analysis

**DOI:** 10.3389/fphar.2025.1652957

**Published:** 2025-10-07

**Authors:** Robert Siegel, Sophie Schlosser-Hupf, Martina Müller-Schilling, Samer A. Kharroubi, André Gessner, Nahed El-Najjar

**Affiliations:** ^1^ Institute of Clinical Microbiology and Hygiene, University Hospital Regensburg, Regensburg, Germany; ^2^ Department of Internal Medicine I, Gastroenterology, Hepatology, Endocrinology, Rheumatology, Immunology, and Infectious Diseases, University Hospital Regensburg, Regensburg, Germany; ^3^ Department of Nutrition and Food Sciences, Faculty of Agricultural and Food Sciences, American University of Beirut, Beirut, Lebanon; ^4^ Department of Pharmacology and Toxicology, Faculty of Medicine, American University of Beirut, Beirut, Lebanon

**Keywords:** drug-drug interactions, cephalosporins, 5-fluorouracil, sepsis, oncology, adverse drug reactions

## Abstract

**Introduction:**

Chemotherapeutic agents, despite their toxicities, variability in individual response, and risk of drug-drug interactions, are essential in the treatment of gastrointestinal cancers. Due to their immunosuppressed state, cancer patients often require concurrent antibiotic therapy, most commonly with cephalosporin antibiotics (CAB) (β-lactams), for treatment or prophylaxis of infections. However, little is known about potential interactions between CAB and antineoplastic agents, such as 5-Fluorouracil (5-FU), Capecitabine, and Trifluridine/Tipiracil.

**Methods:**

This retrospective study aimed to evaluate the impact of CAB therapy on the efficacy and toxicity of these chemotherapeutics in patients treated at the University Hospital Regensburg, Germany. A total of 19 cancer patients receiving CAB were compared with 19 optimally matched controls who did not receive CAB. Matching criteria included age, sex, cancer type, chemotherapy regimen, and cycle interval.

**Results:**

CAB-treated patients experienced a median delay of 6 days in receiving the subsequent chemotherapy cycle, likely reflecting infection-related vulnerability. Despite this, the CAB group demonstrated improved clinical outcomes, with a reduction in tumor progression, an increase in stable disease and tumor regression staging results compared to the control group (p = 0.049). The CAB group also showed a more favorable side effect profile, with milder toxicity despite higher overall medication burden. Notably, when CAB were used alone and for longer durations, side effects remained low.

**Discussion and Conclusion:**

Collectively, concomitant use of cephalosporins with 5-FU, Capecitabine, or Trifluridine/Tipiracil does not impair antitumor efficacy or increase toxicity. Of clinical relevance, CAB therapy enhances clinical outcomes and survival, highlighting the need for further prospective studies on specific antibiotic-chemotherapy interactions.

## Introduction

Drug-drug interactions (DDIs) impact the therapeutic or adverse effects of a single drug or a combination of drugs. Unforeseen DDIs account for 12.5% of events that impede optimal clinical outcomes (Ebbesen et al., 2001). Interestingly, a notable proportion of patient deaths (18.2%) in internal medicine departments were categorized as directly or indirectly linked to the use of one or more drugs. This corresponds to 9.5 deaths per 1,000 hospitalized patients (Ebbesen et al., 2001). Astonishingly, 4% of cancer-related fatalities are attributed to DDIs, making it a significant concern in the healthcare system (Buaj et al., 2001). The increased propensity of cancer patients to DDIs is due to the concomitant administration/reception of different drugs (i.e., chemotherapy, analgesics, and antibiotics) (Ismail et al., 2020). ß-lactams are among the most commonly used antibiotics to manage or prevent infections in immunosuppressed cancer patients (Walsh and Schimpff, 1983). Most patients’ reported DDI data, associated with severe toxicity (Dalle et al., 2002; Jarfaut et al., 2013; Nierenberg and Mamelok, 1983) and enhanced efficacy (Iven and Brasch, 1990; Zhu et al., 2022), result from the concomitant use of several antibiotics with methotrexate (an antineoplastic agent); patients’ data with other antineoplastic agents are still scarce. Yet, ample *in vitro* and *in vivo* studies document the beneficial and detrimental effects of combining other antineoplastic agents and antibiotics. For instance, it has been shown that cefepime and ceftazidime (cephalosporin antibiotics) antagonize the effect of 5-Fluorouracil (5-FU) on colorectal cancer cell lines (Pfab et al., 2022). Interestingly, ceftazidime also demonstrates potent anticancer properties *in vitro*, yet its efficacy is also antagonized by 5-FU (Pfab et al., 2022). Furthermore, 5-FU also exacerbates, in a murine model, the occurrence of convulsive seizures, known side effects associated with cefepime use (Suemaru et al., 2019). Moreover, an intriguing observation reveals that antineoplastic agents can diminish or enhance antibiotics’ efficacy and inhibit bacterial growth (Ueda et al., 1983). Interestingly, while methotrexate reduces cephalosporin activity against certain bacteria (Gieringer et al., 1986), 5-FU shows intrinsic antimicrobial effects (Gieringer et al., 1986; Zhang et al., 2024a) and enhances the activity of selected antibiotics against nosocomial pathogens (Sedlmayer et al., 2021; Niazy et al., 2024). 5-FU also potentiates meropenem (ß-lactam)s’ effects against carbapenem-resistant Gram-negative bacteria isolated from human clinical samples (Zhang et al., 2024b). Interestingly, emerging evidence highlights the potential of 5-FU and its prodrug, Capecitabine, as promising adjuvants for combating bacterial infections while simultaneously targeting tumor metabolism. For example, oral administration of Capecitabine and 5-FU significantly reduced *Staphylococcus aureus* infections and improved survival in mice (McLeod et al., 2021). Interestingly, a combination formulation of 5-FU and Cephem (cephalosporin) has been suggested as a potential prodrug to selectively activate and exert its therapeutic effects against cancer cells within the targeted action region, whether the tumor or infection site (Phelan et al., 2009). It is unsurprising, therefore, that some recent studies highlight the partially effective antimicrobial activity of 5-FU, which could be exploited as a complementary or alternative antibiotic agent for specific pathogens (Zuo et al., 2024). Not only do anticancer drugs have potential antimicrobial activities, but the potential beneficial anticancer effect of antimicrobial agents is also unsurprising, considering their diverse range of possible off-target applications. Several clinical trials are ongoing to evaluate the efficacy of antibiotics against various cancer types (Pfab et al., 2021). Hence, antimicrobial agents offer promising prospects as cost-effective and potent drugs to be repurposed against different cancer types, either as stand-alone treatments or combined with chemotherapy (Pfab et al., 2021). Despite the extensive worldwide use of ß-lactams across various clinical settings to treat different infection types (Chowdhary et al., 2020; Cente rs for Disease Control and Prevention NCfEaZIDN and Division of Healthcare Quality Promotion DHQP, 2022), evidence about their potential benefit or harmful interactions with antineoplastic agents in cancer patients remains inadequate and controversial. Multiple studies highlight a detrimental effect of antibiotics (β-lactam/cephalosporin antibiotics (CAB)) when co-administered with chemotherapeutic or immunotherapeutic agents (Fang et al., 2025; Guerrero et al., 2025; Elkrief et al., 2024). Conversely, other reports suggest a potential clinical benefit when prophylactic antibiotics are initiated concurrently with cancer therapy (Zhang et al., 2025). Unfortunately, predictions indicate a future rise in the incidence of sepsis among cancer patients, particularly alongside an increase in cases of organ failure, necessitating the use of multiple therapies (Te et al., 2020). Given the high risk of poor clinical outcomes, scarce and controversial data about the positive or negative impact of combination therapy between antibiotics and antineoplastic agents make it necessary to evaluate DDIs in cancer patients. Trifluridine/Tipiracil, Capecitabine, and 5-FU are extensively used for treating colorectal or related intestinal cancers, despite their toxicity and the significant intra- and inter-individual variability in responses, as well as the challenging therapeutic interactions associated with their use (Brummett, 1981; El-Najjar et al., 2017; van Leeuwen et al., 2013).

This study evaluates potential DDIs between cephalosporins (β-lactams) and the fluoropyrimidines Trifluridine/Tipiracil, Capecitabine, and 5-FU, a regimen commonly used in the management of gastrointestinal malignancies at the Institute of Internal Medicine, University Hospital Regensburg, Germany.

## Methods

### Trial design and outcome

This study analyzed records of patients with gastrointestinal (GI) cancer treated in the Gastroenterology Department of the University Hospital Regensburg between 2017 and 2023 (Ethical approval No: 25-4221–104).

The goal of this study was to assess the pharmacological interactions between cephalosporins and fluoropyrimidine analogs, specifically Trifluridine/Tipiracil, Capecitabine, and 5-FU, and provide further clinical evidence to support therapeutic decision-making regarding their combined usage.

The primary objective focused on patient outcomes in the context of potential DDIs, particularly concerning 1) tumor progression, 2) antibiotic efficacy and infection control, and 3) the occurrence of treatment-related side effects compared to the single application of the aforementioned antineoplastic agents in the absence of antibiotics.

The secondary objective was to evaluate the influence of additional variables, including cancer type, co-administered chemotherapeutic agents, and other drugs or antibiotics used. These factors were assessed to guide future research and optimize treatment strategies.

It is worth noting, however, that due to the diverse baseline characteristics among patients, investigating the impact of different therapies on life expectancy using a Kaplan-Meier curve proves challenging. Notably, the heterogeneity extends to various cancer manifestations and severities, as well as patients’ lengthy disease histories involving multiple adjustments and changes in chemotherapy regimens across numerous treatment cycles. Consequently, the use of cephalosporin antibiotics (CAB) over short durations, ranging from a few days to 2 weeks, is unlikely to have a discernible impact on the overall disease trajectory, which spans several years and affects survival.

Considering these factors, this study’s primary focus aimed to examine the short to medium-term effects, including CAB performance in treating or preventing septic complications, subsequent staging results to gauge anticancer efficacy, and the occurrence of side effects during ongoing chemotherapy.

Additionally, the study explored the influence of the standard treatment routine within the context of chemotherapy cycles.

### Participants and treatment

The study included adult patients undergoing chemotherapy and receiving regimens containing 5-FU, Capecitabine, or Trifluridine/Tipiracil, such as FOLFOX (a 5-FU-containing regimen), FOLFIRI (a 5-FU-containing regimen), or CAIRO (a Capecitabine-containing regimen). These patients were also treated with CAB during the intermittent application period. The patients had a confirmed diagnosis of intestinal cancer. This included malignancies located in the colorectal region, esophagus, pancreas, stomach, and intrahepatic bile ducts (cholangio-cellular carcinoma). Cancer patients receiving CAB prophylactic treatment were analyzed separately from patients receiving antimicrobial therapy to manage an infection. For the reference group, we scanned the same data collection for patients receiving the identical chemotherapy regimen in the same application cycle without antibiotic co-treatment. From the 83 possible matching patients, it was possible to identify the most suitable patient group based on age, sex, cancer manifestation, and progression. The chemotherapy regimens were administered in cycles lasting 2–4 weeks, with dosage adjustments based on the cancer presentation and tolerance to side effects. Data collection involved reviewing unplanned hospitalization records, staging results and evaluations, and documenting the course of therapy during each treatment session. This documentation included recurring laboratory results, precise medication doses, and patient feedback on side effects.

Concerning CAB administrations, while Ceftriaxone is limited to parenteral use, the other cephalosporins (Cefpodoxime, Cefuroxime, and Cefixime) are also available orally, which enables outpatient antimicrobial therapy after discharge. The low number of patients receiving CAB as prophylaxis stems from the fact that, according to the antibiotic stewardship team, prophylaxis is not recommended unless for a specific purpose or indication, such as perioperative procedures. Therefore, these patients were excluded from the analysis.

### Statistical analysis

Data analysis was performed using SPSS version 29.0 (IBM). Descriptive statistics were used to present the data. Categorical variables are reported as counts and percentages, while continuous variables are expressed as either the mean ± standard deviation or the median [interquartile range], depending on the distribution. For group comparisons, the t-test or Mann–Whitney U test was applied for continuous variables, and the Chi-square test or Fisher’s exact test for categorical variables, based on sample size and expected frequencies. Standard deviation quantified dispersion around the mean, whereas the median, as a robust measure of central tendency, reduced the influence of outliers. All tests were two-tailed, with p-values ≤0.05 considered statistically significant.

## Results

### Study design

This study (2017-2023) evaluated the clinical outcomes of patients with cancer of the GI tract receiving chemotherapy containing 5-FU, Capecitabine, and Trifluridine/Tipiracil compared to a matching group who received CAB as a therapeutic regimen to eradicate infection. Data was compared regarding tumor progression, side effects, interactions with the chemotherapy regimen, chemotherapy dosage, and CAB treatment duration. In the present study, additional statistical covariate-balancing techniques, such as propensity score matching or weighting, were not applied due to the small sample size and heterogeneous patient histories, which are challenging to implement and can limit the feasibility and stability of such methods. Instead, *a priori* manual matching approach based on key prognostic and treatment-related variables was used, enhancing comparability while avoiding overfitting or instability associated with automated balancing algorithms in small datasets. From the 83 patients in the non-CAB chemotherapy cohort, 19 were selected as the reference group through a predefined manual matching process. Priority was first given to identifying patients who had received the identical chemotherapy regimen and cycle intervals as those in the CAB group. Next, cancer type and stage were matched by grouping closely related entities, such as gallbladder and cholangiocellular carcinoma, when exact matches were not available due to their similar anatomical origins, clinical behaviors, and treatment approaches. Finally, it was essential to align the age and sex distribution as closely as possible between the groups. This stepwise selection ensured that each CAB patient was matched to a reference patient with the most comparable clinical and treatment profile, thereby minimizing baseline differences between groups despite the small sample size. A 1:1 matching strategy was employed to optimize comparability between the CAB and reference groups. This approach ensured balanced baseline characteristics and allowed for parallel and synchronous observation periods, which is particularly important for time-sensitive outcomes and dynamic clinical endpoints. Unlike higher matching ratios (i.e., 2:1), the 1:1 design avoided weighting bias, enabled control of confounding variables, and preserved statistical validity, especially given the limited sample size. It also allowed for consistent observation intensity across groups, enabling a more accurate evaluation of CAB-specific effects. Collectively, among the 107 analyzed patients receiving chemotherapy containing Trifluridine/Tipiracil, Capecitabine, or (mostly) 5-FU, five received CAB as prophylaxis, nineteen cancer patients received CAB for infection treatment, and 83 received only the chemotherapy regimen. From the latter reference group (83 patients), nineteen were identified as optimal matches based on the criteria described above. The CAB therapy included mainly ceftriaxone, cefixime, cefpodoxime, and cefuroxime. Since the number of patients receiving CAB as prophylaxis is low (5 patients only), these patients were excluded from the analysis. In the following, nineteen cancer patients undergoing CAB treatment were compared to a reference group (n = 19) receiving chemotherapy but not CAB. Further analysis revealed no significant difference [reference group (62.5 ± 11.72) vs. CAB group (63 ± 8.79) years, p > 0.5, Chi-square test] in age distribution, with both groups comprising eight patients older than 65 years and eleven patients younger than 65 years (Table 1). Furthermore, despite the higher percentage of male patients in the CAB group [CAB group (32%) vs. reference group (16%)] (Table 1), this was not statistically significant (p > 0.5, Chi-square test) (Table 1). As shown in Table 1, no significant differences were found between the CAB and reference groups (p ≥ 0.05, Chi-square test and Fisher’s exact test) regarding various additional characteristics of the study population, including cancer type, chemotherapy basis regimen, CAB therapy, and whether the patients received CAB therapy in the last months. It is worth noting that colorectal cancer represented the largest proportion of cases (45%), followed by pancreatic (24%), esophageal (21%), and stomach cancer (5%). These major tumor entities were equally distributed between the CAB and reference cohorts, ensuring comparability. Gallbladder and cholangiocellular carcinomas were grouped based on their anatomical proximity, common biliary origin, and comparable clinical behavior and therapeutic approaches.

**TABLE 1:** Characteristics of the study population. The different population groups are presented with the best matching requirements in medication and comparability. Patients of the comprehensible reference group received no CAB medication; respective boxes to the missing antibiotic treatment were marked “N/A”.

		Total (N = 38)	References (N = 19)	CAB (N = 19)	P-value
Age, N [%]	≥65 years	16 [42.1]	8 [42.1]	8 [42.1]	1**
Gender, N [%]	Men	29 [76.3]	16 [84.2]	13 [68.4]	0.447
Tumor location, N [%]	Colorectal	17 [44.8]	9 [52.9]	8 [47.1]	1.000
	Pancreas	9 [23.7]	4 [21.1]	5 [26.3]	1.000
	Esophagus	8 [21.1]	4 [21.1]	4 [21.1]	1**
	Stomach	2 [5.3]	1 [5.3]	1 [5.3]	1**
	Gall bladder/Cholangiocellular carcinoma	2 [5.3]	1 [5.3]	1 [5.3]	1**
Tumor locations combined					0.997
Cancer extent, N [%]	Metastasis	24 [63.2]	11 [57.9]	13 [68.4]	0.737
Chemo regimen basis, N [%]	5-FU	34 [89.5]	17 [89.5]	17 [89.5]	1**
	Capecitabine	2 [5.3]	1 [5.3]	1 [5.3]	1**
	Trifluridine/Tipiracil	2 [5,3]	1 [5.3]	1 [5.3]	1**
Cephalosporin, N [%]	Ceftriaxone	N/A	N/A	13 [68.4]	N/A
	Cefuroxime	N/A	N/A	4 [21.1]	N/A
	Cefixime	N/A	N/A	1 [5.3]	N/A
	Cefpodoxime	N/A	N/A	1 [5.3]	N/A
Antibiotics used in last 3 months		11 [28.9]	5 [26.3]	6 [31.6]	1.000
Comorbidities		19 [50]	6 [31.6]	13 [68.4]	0.050

The numbers within the brackets represent the percentage shares for the respective number of patients in the corresponding row.

The values marked with “**” refer to the search for a chemo-identical reference group and, by that, identical values.

The p-value indicates the success of matching between the reference and CAB groups. A high p-value indicates a near-identical distribution. Matching was not performed for comorbidities and antibiotics used in the last 3 months; these data were retrospectively collected as part of potential supplementary analyses.

The statistical tests used were the Chi-square test and Fisher’s exact test.

Only comorbidity rates were significantly higher in the CAB group (p = 0.050, Chi-square test). One possible explanation is the higher incidence of infections and sepsis, necessitating the use of CAB antibiotics (Drijkoningen and Rohde, 2014; Nudel et al., 2019). In summary, aside from comorbidities, the participants in the reference group were well matched with those in the CAB group.

### CAB group patients require additional time before receiving their second chemotherapy cycle

**FIGURE 1:**
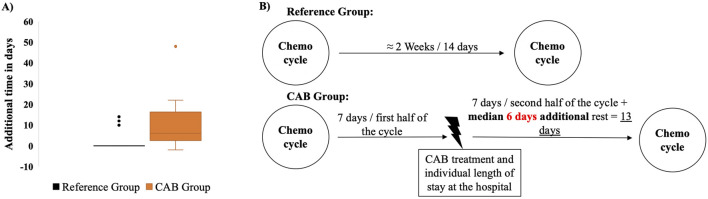
The difference between the time gaps between chemotherapy cycle → Infection and infection → chemotherapy cycle **(A)** and the scheme explaining how the extra recovery time is determined **(B)**. **(A)** This box plot calculates the difference between the two-time gaps with a median of 6 days and the maximums at −2 and 48 days in the CAB group. By only three values deviating from 0 in the reference group, there is no change in the median from 0. With an SD ± 95%, p = 0.011 ≤ 0.05, t-test, the second time gap is significantly longer than the first. **(B)** By assuming the onset of infection in the middle of the cycle, patients took a median of 6 days additional to their usual interval to recover in the second half of the cycle after infection and stationary treatment to receive the next chemotherapy application.

Considering the patients’ short chemotherapy cycle intervals (1–4 weeks), the variable individual onset of the infection, different courses of antimicrobial therapy that varied according to the type of infection, and the different lengths of stay in the hospital, the start of infection, is assumed for simplicity, in the middle of the gap between the two cycles. This allowed comparison of recovery durations between treatment cycles. The difference was expressed as either an extended or shortened recovery time relative to the actual cycle length. A comparison of the courses taken by the reference and CAB groups shows that CAB patients, in addition to the hospitalization time, required a median of 6 days longer after infection to receive their next chemotherapy cycle, compared to the reference group (Figure 1A). Notably, in all but one patient, the interval between CAB treatment and the subsequent chemotherapy cycle exceeded the typical cycle length, possibly reflecting the need for extended recovery due to increased vulnerability resulting from both the infection and the CAB therapy. To simplify the calculation, a scheme explaining how the extra recovery time is estimated, assuming a chemotherapy cycle interval of 2 weeks in the example, is presented in Figure 1B.

Table 2 provides an extensive overview of the chemotherapy regimen before and post-infection, the period between chemotherapy and CAB therapy, and the required time between the end of infection and the following chemotherapy. Unfortunately, two out of 19 patients died due to sepsis, and a single patient decided not to continue with the next cycle of chemotherapy. Moreover, two patients received their CAB simultaneously with chemotherapy. Interestingly, the data shows that, except for one patient, all patients’ second-period gap between CAB and chemotherapy is longer than their usual chemotherapy cycle gap, evidenced by a positive value at the additional rest to usual chemotherapy cycles (Table 2).

**TABLE 2:** Effect of CAB therapy on chemotherapy. The recovery time between chemotherapy cycles regarding the continuation of usual chemotherapy, sorted by cancer type, around infection in the CAB group, is compared with the identical time intervals in the reference group. With a calculated occurrence of infection in the middle of the chemotherapy interval, the assumed time to resumption before infection, in the ideal case, should be equal to or shorter than the time after termination of measures and infection until the resumption of the next interval. The tab’s additional rest is calculated by subtracting these two time periods. The calculated value indicates the physical stress and associated recovery capacity around the infection events. Changes in the chemotherapy regimen are also considered. Since two patients in the CAB group died, and one stopped chemotherapy, no data are available regarding the time course and are marked with “N/A”. One patient in the CAB group could continue his chemotherapy earlier than the estimated time and, therefore, could achieve a negative number. There were no changes in the chemotherapy regimen or time course deviations in all empty fields.

References group					CAB group				
Cancer type	Chemotherapy before infection or equivalent cycle in reference	Change of chemotherapy or composition	Reason for the change in the chemotherapy	Additional rest to the usual chemotherapy cycles	Cancer type	Chemotherapy before infection or equivalent cycle in reference	Change of chemotherapy regimen or composition	Reason for the change in the chemotherapy	Additional rest to the usual chemotherapy cycles
Colorectal	FOLFOX	Change to FOLFIRI	Staging results, tumor progression		Colorectal	FOLFOX	Escalation on 5-FU dose from 50% to 75%	Staging results, tumor progression	14
Colorectal	FOLFIRI				Colorectal	FOLFIRI	Addition of regorafenib	Staging results, tumor progression	1
Colorectal	FOLFOX				Colorectal	FOLFIRI			4
Colorectal	FOLFOX				Colorectal	FOLFIRI			N/A
Colorectal	FOLFIRI			14	Colorectal	FOLFOXIRI	Change to CAIRO	Side effects	−2
Colorectal	FOLFIRI	Escalation on 5FU dose from 80% to 90%			Colorectal	FOLFOX			2
Colorectal	FOLF				Colorectal	FOLF			20
Colorectal	FOLFOXIRI				Colorectal	Trifluridine/Tipiracil	Change to FOLFIRI	Staging results, tumor progression	5
Colorectal	Trifluridine/Tipiracil								
					Pancreas	FOLFIRINOX			6
Pancreas	FOLFIRINOX				Pancreas	FOLFIRINOX	Reduction on 5-FU dose from 100% to 75%	Side effects	6
Pancreas	FOLFIRINOX	Reduction on 5FU from 100% to 75%		10	Pancreas	FOLFIRINOX			8
Pancreas	FOLFIRINOX				Pancreas	FOLFOX			6
Pancreas	FOLFIRINOX				Pancreas	FOLFIRINOX	Change to Gemcitabine mono	Staging results, tumor eradication	1
Esophagus	FOLFOX				Esophagus	FLOT	Change to FOLFOX	Staging results, tumor progression	48
Esophagus	FLOT				Esophagus	C/F			17
Esophagus	C/F				Esophagus	FLOT			N/A
Esophagus	FLOT			12	Esophagus	FOLFOX			N/A
Stomach	FLOT	Reduction on 5FU from 100% to 75%			Stomach	FLOT			22
Cholangiocellular carcinoma	FOLFIRI				Gall bladder	FOLFIRI			4

Abbreviations: FOLFIRI, folinacid, 5-FU, irinotecan; FOLFIRINOX, folinacid, 5-FU, irinotecan, Oxaliplatin; FOLFOX, folinacid, 5-FU, oxaliplatin; FOLF, folinacid, 5-FU; FLOT, folinacid, 5-FU, oxaliplatin, Docetaxel; C/F = capecitabine, 5-FU; CAIRO, capecitabine, Irinotecan, Oxaliplatin; G emcitabine = Pyrimidine analog.

### Clinical outcome of chemotherapy in patients who received CAB therapy is more favorable compared to the reference group

Gastrointestinal carcinomas are complex and require close monitoring of the chemotherapeutic dosage to ensure timely and positive effects. Depending on the aggressiveness of the diagnosis, staging results are usually re-evaluated every few months to 6 months. During one of these intervals, an infection requiring CAB treatment occurred. Due to the close monitoring of tumor development, staging evaluation is considered an adequate method for assessing the therapy’s antitumor effect. This is interesting, as it provides insight into whether CAB therapy has a beneficial or detrimental impact on staging outcomes within the context of the investigated DDI. Therefore, the effect of CAB treatment on the earliest possible staging results, used as a primary objective indicator of antitumor efficacy, was compared between the CAB and reference groups.

Table 3 summarizes the staging results of the different tumors in the CAB and reference groups. Unfortunately, no data on staging results could be found for three patients in the CAB group; these patients might have terminated their clinical treatment and diagnostic in the oncology department at their request, changed their place of living or hospital altogether, or died privately under the guidance of a general practitioner without documentation in the hospital’s files. Nevertheless, the subsequent staging results of the remaining 16 patients were analyzed using available data to assess the impact of the CAB treatment in the CAB group, along with chemotherapy, compared to the reference group receiving only chemotherapy.

**TABLE 3:** Staging results of the different tumors in CAB and reference groups. The staging results are intended to give the best possible impression of cancer development and, at the same time, to evaluate the antitumor effect of CAB therapy. The latest staging results were assessed after the infection. The results are listed regarding progress, stable disease, or regression. Further aspects were the diagnosis, the metastatic state, and the chemotherapy regimen. The precise grading of the diagnosis is neglected and simplified in terms of whether metastasis occurs.

Chemotherapy	Cancer type	Staging post antibiotic application	Chemotherapy	Cancer type	Staging post antibiotic application
Tumor progression					
References group			CAB group		
FOLFOX	Colorectal	Multiple small circular foci, tumor progression	FOLFIRI	Colorectal	Local progression, progressive pulmonary and nodal (lymphatic) metastasis, tumor progression
FOLFOX	Colorectal	New metastases, tumor progression	FOLF	Colorectal	Recurrence of lung metastases, tumor progression
FOLFIRI	Colorectal	Progress of metastatic burden, tumor progression	Trifluridine/Tipiracil (Lonsurf)	Colorectal	Progress of hepatic, pulmonary, osseous filialization and metastasis, tumor progression
FOLFIRI	Colorectal	Increase in lesion size, tumor progression	FOLFIRI	Colorectal	Progress pulmonary, hepatic, and lymphatic metastases, tumor progression
FOLFOXIRI	Colorectal	Tumor progression			
FOLF	Colorectal	Progression of tumor burden, tumor progression			
Trifluridine/Tipiracil (Lonsurf)	Colorectal	Size progressive metastases, tumor progression			
FOLFIRI	Cholangiocellular	Size progressive metastatic liver nodes, tumor progression			
FOLFIRINOX	Pancreas	Size progressive tumor, tumor progression			
FLOT	Esophagus	New suspect loci and size progression, tumor progression			
FLOT	Stomach	Growth of tumor manifestation, tumor progression			
FOLFIRINOX	Pancreas	Tumor progression			

Tumor regression					
References group			CAB group		
FOLFOX	Esophagus	Size decreasing metastatic nodes, tumor regression	FOLFIRINOX	Pancreas	No evidence of local recurrence or thoracoabdominal branching, end of chemo
FOLFIRINOX	Pancreas	Size reduction of tumor, tumor regression	FOLFOX	Pancreas	Unobtrusive findings without local recurrence or branchialization
FLOT	Esophagus	Reduction of metastases, tumor regression	FLOT	Stomach	Size-responsive liver and lymph node metastases
C/F	Esophagus	Size responsive metastases, tumor regression	FOLFIRINOX	Pancreas	Slightly regressive size, tumor markers strongly declining

Stable disease					
References group			CAB group		
FOLFOX	Colorectal	Stable disease	FOLFOX	Colorectal	Stable disease
FOLFIRI	Colorectal	Stable disease	FOLFOX	Colorectal	Stable disease
FOLFIRINOX	Pancreas	Stable disease	FOLFOXIRI	Colorectal	Stable disease
			FOLFIRI	Gall bladder	Stable disease
			FOLFIRINOX	Pancreas	Stable disease
			C/F	Esophagus	Stable disease

Death					
			CAB group		
			FLOT	Esophagus	Death
			FOLFIRI	Colon	Death

**FIGURE 2:**
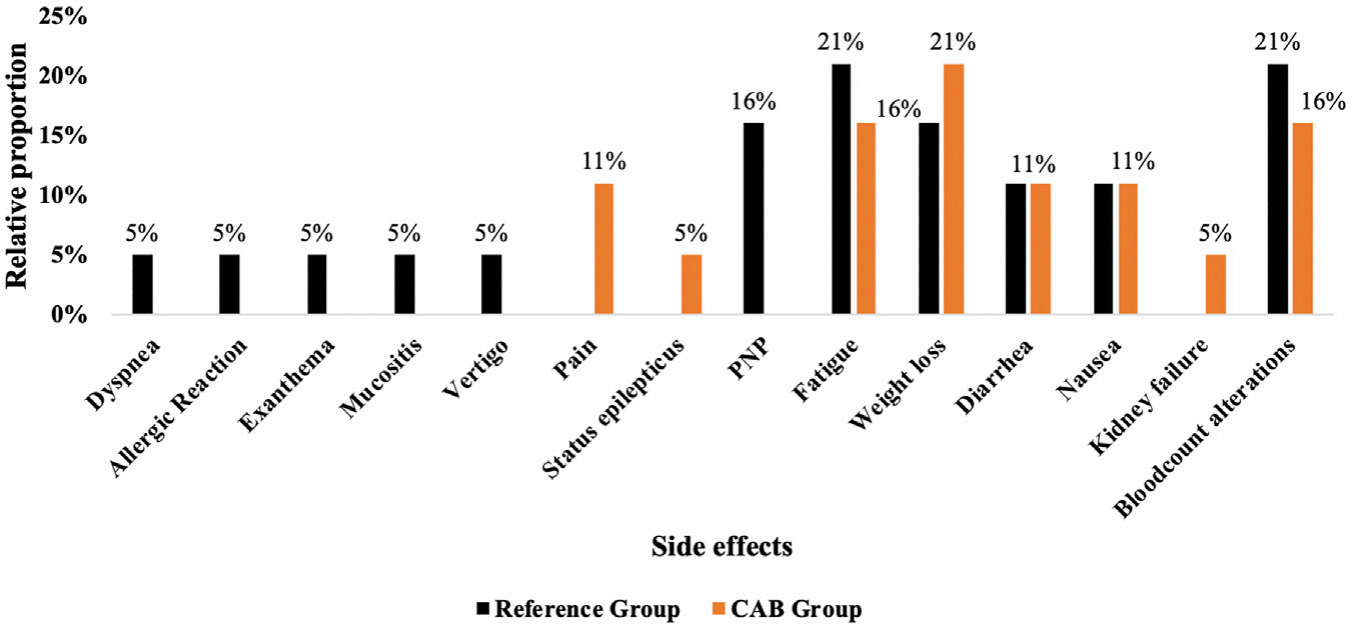
Staging results **(A)** and the distribution of staging results on cancer types **(B)**. **(A)** A basic percentage overview of the staging results compared to the Reference group (in black) and the CAB group (in orange). The tab “Death” is excluded because it is more of an infection course than an antitumor result. With an SD of ±95%, p = 0.049, Chi-square test, the CAB group demonstrated a significantly superior outcome, characterized by a higher proportion of patients with the desired tumor regression or stable disease and a lower proportion with tumor progression. **(B)** A general breakdown of staging results by cancer type. The data was categorized based on the diverse initial manifestations of tumor diseases and their progression. It was organized according to staging outcomes, including progress, stable disease, regression, and, in the case of infection, death. Unfortunately, staging data for three patients in the CAB group could not be obtained. It is possible that these individuals succumbed to the infection in a different medical facility or privately, or chose to discontinue all treatment at their request, refusing to divulge any information or data.

The staging results were categorized into tumor progression, stable disease (a favored outcome), and tumor regression (the most favorable or optimal outcome). Given the limited sample size in both groups (n = 19 per cohort), subdividing the staging results into three separate categories (tumor progression, stable disease, tumor regression) in a Chi-square test reduces the expected cell counts in several cells below the recommended threshold, which diminishes statistical power and makes the test results unstable. To maintain adequate statistical validity and interpretability while avoiding sparse data bias, “stable disease” and “tumor regression” were combined into a single “favorable outcome” category and compared against “unfavorable outcome” (tumor progression). This dichotomization increases the number of observations per cell, preserves the clinical distinction between good and poor responses, and allows for a more robust statistical comparison between groups. Figure 2A shows that the CAB group had a more favorable prognosis than the reference group, with a 34 percentage point reduction in tumor progression, a 22 percentage point increase in stable disease, and a 12 percentage point increase in tumor regression (p = 0.049, Chi-square test). It is worth mentioning, however, that the data represent the effect of CAB therapy on the total number of cancers identified in both groups (Table 1). By dissecting individual tumor manifestations and their respective outcomes, focusing notably on esophageal, stomach, pancreatic, and colorectal diagnoses, it seems that the regression of pancreatic cancer in the CAB group is substantially higher than the regression observed in the reference group (Figure 2B). When stratified by tumor type, patients with colorectal and pancreatic cancer in the CAB group more often showed favorable outcomes (stable disease or regression) compared with the reference group, where tumor progression predominated. In contrast, esophageal cancer tends to have a favorable outcome in the reference group. Yet, just as for gastric and biliary tract cancers, interpretation is limited by the small number of patients (Table 1). Unfortunately, the individual group sizes are too small for statistical analysis of abnormalities and significance. Larger-scale future studies are warranted in this regard.

### Patients with CAB treatment tend to have fewer and milder side effects

**FIGURE 3:**
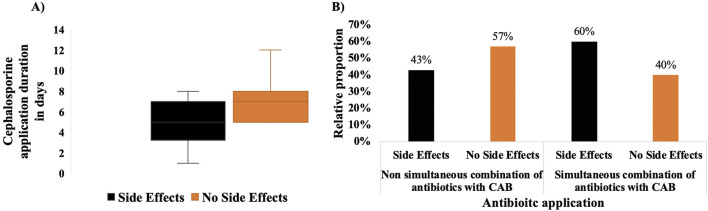
Distribution of side effects in CAB and reference groups. All the side effects that occurred are listed and compared. The reference group is shown in black and the CAB group in orange. Adverse effects were recorded individually. Some patients experienced none, while others developed multiple different side effects. The percentage values were calculated based on the number of patients in each group. Consequently, it is possible that an equal number of patients in both groups experienced adverse effects; however, the total number of adverse events was higher in the reference group than in the CAB group. The well-known hand-foot syndrome associated with 5-FU therapy could not be reliably identified from the available documentation. To avoid misinterpretation, all related symptoms were consolidated under the ‘polyneuropathy (PNP)' category for standardized classification. Furthermore, the occurrence of Exanthema may also be in connection with hand-foot syndrome. With an SD ± 95%, p = 0.589, Chi-square test, no statistically significant difference was observed between the two groups regarding the distribution of side effects.

To assess the antibiotic’s effectiveness within the context of combined therapy involving 5-FU, the impact of the infection in terms of side effects, duration of CAB administration, drug combinations, and subsequent ability to resume the usual chemotherapy cycle was evaluated. Interestingly, despite the increased potential for more side effects due to the infection, the less stable health condition, and the increased drug consumption and variety in the CAB group, an examination of acute side effects during the subsequent chemotherapy cycle revealed that 63% of individuals in both groups experienced multiple and diverse side effects. However, by ruling out kidney failure, which appeared based on the diagnosed usage of Vancomycin and not CAB, the CAB group exhibited even marginally fewer side effects. Compared to the CAB group, a broader array of side effects was seen in the reference group. For instance, only patients in the reference group reported well-known chemotherapy-associated complaints, including mucositis and oropharyngeal candidiasis (Soor, oral candidiasis) (Diaz et al., 2019). Surprisingly, upon directly comparing side effects, it was observed that classic adverse effects associated with 5-FU therapy, such as polyneuropathy or dysesthesia and paresthesia related to hand-foot syndrome (referred to as “PNP”), were absent in the CAB group (Figure 3). Furthermore, other side effects associated with chemotherapy, such as vertigo, dyspnea, allergic reactions, mucositis, and exanthema, appear to be absent in the CAB group (Figure 3). Interestingly, the groups also have variations in blood counts (thrombocytopenia, neutropenia, or leukocytosis). Notably, while leukocytosis was similar in both groups and thrombocytopenia was only present in the CAB group, compared to the reference group, neutropenia associated with the chemotherapy was markedly decreased in the CAB group (Table 4). Surprisingly, gastrointestinal side effects, such as fatigue, weight loss, nausea, and diarrhea, which can also be attributed to the infection and heavy antibiotic demand and consumption, were not worse in the CAB group compared to the reference group. The conditions seemed stable between groups, with the absence of difference between the incidence of diarrhea and nausea, and only a slight change in fatigue (5 percentage point decrease vs. the reference group) and weight loss (5 percentage point increase vs. the reference group). The occurrence of status epilepticus (a life-threatening condition of continuous or rapidly recurring seizures) as a neurological response in the CAB group is not surprising, considering the known association of CAB and other antibiotics with unfavorable drug reactions, particularly neurotoxicity (Cock, 2015; Maan et al., 2022; Misra et al., 2013). Likewise, side effects affecting patients’ wellbeing, such as pain, can also be related to infection and clinical stay. For instance, infections, especially in immunocompromised patients, often lead to inflammatory responses, which can cause pain in various tissues or organs (Chen et al., 2018). Furthermore, antibiotic usage is also associated with infection-related pain (Chen et al., 2018). The presence of pain only in the CAB groups shows that the presence of infection and antibiotic usage can exacerbate infection-related pain in cancer patients (Cohen et al., 2022).

**TABLE 4:** Investigation of the antibiotic and anti-infectious effect. The infection itself, the antibiotics used, the side effects in the matched reference and CAB groups, and the ability of CAB patients to proceed with their usual next planned chemotherapy cycle were analyzed and compared. Patients in the Reference group were listed 1:1 with CAB patients based on the chemotherapy regimen to which they were matched. The two patients who died and the one who decided to discontinue chemotherapy are marked with “*” due to the absence of data on subsequent treatment cycles. Regarding the duration of the CAB application, a “+” is used when the available documentation does not specify the exact end date of the CAB treatment. In such cases, the smallest confirmed application duration is reported.

Numbering	Infection	Chemotherapy application on the next possible date	Skipping a cycle of chemotherapy application due to infection fatigue	CAB	CAB application duration	Other antibiotics used	Side effects after the next chemotherapy application	Side effects of matching References patients
1	UTI	x		Ceftriaxone	4-5d	In combination with Vancomycin	Kidney failure under Vancomycin therapy	Fatigue, neutropenia, nausea
2	UTI	x		Cefpodoxime	7d+	In combination with Unacid, Amoxicillin/Clavulanic acid		
3	UTI	x		Ceftriaxone	8d	In combination with Vancomycin		PNP
4	UTI	x		Ceftriaxone	12d	First Pivmecillinam, then a combination of Ceftriaxone and Vancomycin		
5	Port infection	x		Ceftriaxone	5d	Changed to Ciprofloxacin		PNP
6	Port infection	*	*	Ceftriaxone	5d+	Changed to Piperacillin/Tazobactam and vancomycin, then changed to Meropenem, Linezolid and Caspofungine	Weight loss	
7	Port infection	x		Ceftriaxone	6d	Changed to Levofloxacin	Nausea, fatigue	Diarrhea
8	Soft tissue infection via port		1 cycle off	Cefuroxime	7d		Diarrhea	Mucositis and soor, weight loss
9	Abscess	x		Ceftriaxone	5d	In combination with Metronidazole		Exanthema, an anaphylactic reaction
10	Abscess		1 cycle off	Cefuroxime	8d+		Fatigue, bloody diarrhea, weight loss, and respiratory infection	Fatigue
11	Small bowel perforation	x		Ceftriaxone	3d	In combination with Metronidazole	Weight loss	Leukocytosis, neutropenia, fatigue
12	Cholangitis		1 cycle off	Cefuroxime	5d+	In combination with Vancomycin, then changed to Piperacillin/Tazobactam and Vancomycin	Thrombozytopenia	dyspnea
13	Cholestasis	x		Ceftriaxone	7d	Changed afterwards to Piperacillin/Tazobactam, then to Amoxicillin/Clavulanic acid	Painkiller increase	
14	Choledocholithiasis, Cholangitis		1 cycle off	Ceftriaxone	8d	In combination with Metronidazole		PNP, diarrhea, Fatigue, vertigo
15	Meningeosis carcinomata	*	*	Ceftriaxone	4d	In combination with Amoxicillin and Aciclovir	Death, Status epilepticus	
16	Unknown infect		1 cycle off	Ceftriaxone	3d	In combination with Metronidazole, then changed to Piperacillin/Tazobactam	weight loss	Fatigue, nausea, weight loss
17	Unknown infect		1 cycle off	Cefuroxime	1d+	At first Ciprofloxacin then changed to Cefuroxime	Fatigue, nausea, painkiller increase, lasting UTI	
18	Unknown infect	*	*	Cefixime	5d		Death	
19	Unknown infect		1 cycle off	Ceftriaxone	7d	Piperacillin/Tazobactam at first then changed to Cefixime then change to Meropenem and Vancomycin	Neutropenia and Leukocytosis	Weight loss, neutropenia

Abbreviations: UTI, urinary tract infection; Overall pain (infection or inflammation related) is evidenced by the patient’s increasing request for a painkiller.

Table 4 summarizes the data for the CAB group regarding the type of infection, antibiotics used, duration of antibiotic use, chemotherapy regimen, side effects following the chemotherapy regimen, and side effects in the matching reference group.

**FIGURE 4:**
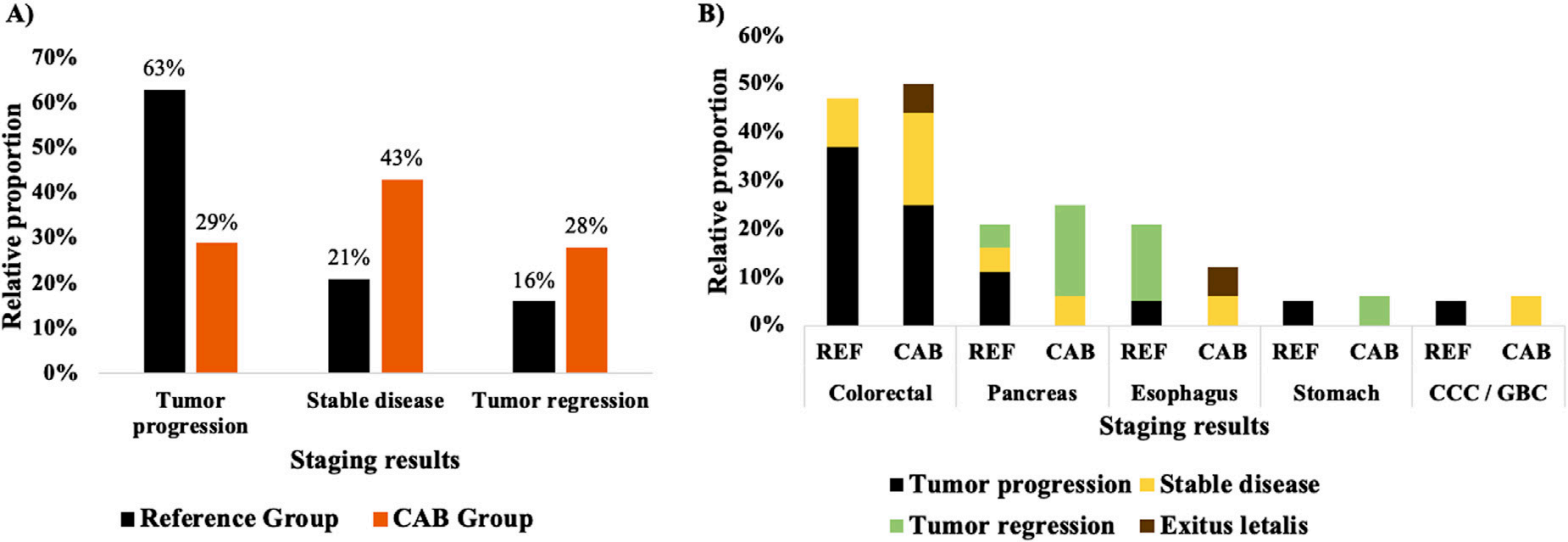
The relationship between cephalosporine treatment duration and the appearance of side effects **(A)** and the distribution and occurrence of side effects associated with antibiotic use **(B)**. **(A)** The appearance of side effects or not in connection with the duration of a CAB application is investigated. With an SD ± 95%, p = 0.033 ≤ 0.05, t-test, the prolonged application of CAB has a significant favorable effect on the appearance of side effects. **(B)** The occurrence of side effects was analyzed in relation to different modes of CAB administration. Given the small number of patients receiving CAB alone (3 out of 19), the groups were stratified to facilitate a better comparison between simultaneous and non-simultaneous use of CAB alongside other antibiotics regarding the occurrence of side effects.

Interestingly, as shown in Figure 4A, the duration of CAB application demonstrated a statistically significant (p < 0.05, t-test) impact on the occurrence of side effects. Patients experiencing side effects received CAB for an average of 5 days, whereas those without side effects received CAB for 7 days. Hence, a more prolonged CAB application has significantly fewer side effects. Since antibiotics are often administered in various combinations, combined or alternating, to achieve a better response by covering a broader spectrum of bacteria and overcoming resistance, we opted to investigate the effect of the CAB therapy alone or with other classes on the occurrence of side effects. Figure 4B illustrates the modes of CAB administration, either as monotherapy or in combination with other antibiotics. Given that only 16% (3 of 19 patients) received CAB exclusively, preliminary observation for monotherapy was limited, prompting a focus on combined antibiotic regimens involving CAB. Within these, two strategies were identified: a simultaneous administration of CAB with multiple antibiotics (i.e., with Metronidazole) and an alternating approach, where CAB was administered briefly before switching to other antibiotics. Treatment success, measured by the absence of side effects, was higher in patients who received CAB more sustainably or intensively. Patients treated with an alternating CAB regimen experienced fewer side effects (57%, four of seven patients) than those who received CAB concurrently with multiple antibiotics (40%, four of 10 patients). Although limited by sample size, this finding suggests that a more targeted or temporally structured CAB application is associated with improved tolerability and potentially more favorable clinical outcomes.

## Discussion

### Cancer and chemotherapy-related infection risk

Cancer patients are more prone to infections and are often treated with antibiotics as part of prophylactic or therapeutic regimens (Mac et al., 2019). Patients’ susceptibility to infection is directly proportional to the degree of impairment in their immunological function. It is widely acknowledged that patients undergoing radiation therapy and cytotoxic drug treatments face an elevated risk of fatal infections (Campbell et al., 1973; Harris et al., 1976). Moreover, studies have shown that 5-FU can induce septicemia in mice, particularly from *Klebsiella pneumoniae* and *Escherichia coli* (Hirakata et al., 1996), especially following bowel decontamination and inoculation with resistant strains. The risk is heightened by factors such as tumor-related obstruction, advanced age, invasive procedures, indwelling catheters, and cancer-related immunosuppression (Campbell et al., 1973; Harris et al., 1976). This latter case can arise from the underlying cancer and therapeutic interventions employed (Frumento et al., 2006). Unfortunately, infections frequently arising as a notable adverse consequence of cancer therapy significantly influence the prognosis and quality of life of affected patients and force antibiotic intervention (Åttman et al., 2015).

### Therapeutic challenges of Co-Medications

Integrating antimicrobial and antineoplastic therapies presents therapeutic opportunities and risks due to potential side effects and DDIs. Antibiotic-drug interactions are less studied due to the short duration of antibiotic use, but have significant clinical implications. These interactions can alter drug pharmacokinetics or pharmacodynamics, leading to treatment failure, toxicity, or resistance. These interactions can also be exacerbated by polypharmacy (i.e., a cocktail of additional drugs), patient characteristics, and drug properties, which further complicate the situation.

### Study motivation and clinical context

Despite the divergent perspectives on the concurrent administration of 5-FU, Capecitabine, and Trifluridine/Tipiracil in combination with CAB therapy, ranging from their proposed therapeutic benefits to concerns regarding their adverse interactions, they are still widely used. Therefore, this study retrospectively explored the clinical implications of combined CAB therapy in cancer patients receiving 5-FU, Trifluridine/Tipiracil, and Capecitabine within the oncology day clinic of the University Hospital Regensburg, Germany. Beyond their common clinical applications, 5-FU, Capecitabine, and Trifluridine/Tipiracil share a mechanism of action that disrupts nucleic acid synthesis, particularly DNA and RNA metabolism, thereby inhibiting cancer cell growth and proliferation (Dubin et al., 2016).

### Study objective

The primary objective was to compare patients receiving 5-FU-, Capecitabine-, or Trifluridine/Tipiracil -based chemotherapy with concurrent CAB therapy to a matched reference group receiving chemotherapy alone, focusing on tumor progression, infection control, and adverse events. A secondary objective was to assess the influence of potential effectors, including cancer type, co-administered chemotherapeutic agents, and specific antibiotics, on patient outcomes. It is worth noting, however, that due to the small sample size of the current retrospective study, highly significant results and broadly generalizable findings must be interpreted with caution. Future studies with larger cohorts are warranted.

### Improved cancer outcomes in the CAB group despite treatment delays

Notably, patients in the CAB group showed better overall cancer outcomes than the reference group, with less tumor progression, higher rates of stable disease, and more frequent tumor regression (p = 0.049) (Figure 2A).

While this trend was consistent across most cancer types, outcomes in colorectal cancer were relatively similar between both groups, and in esophageal cancer, the reference group exhibited slightly better results. Achieving such results, despite the delayed resumption of the subsequent chemotherapy (Figure 1) in the CAB group by a median of 6 days longer than the reference group, suggests a highly positive response regarding cancer disease development to chemotherapy combined with CAB. The obtained results in the CAB group can be due to the anticipated DDIs affecting antitumor outcomes and/or the mutual influence of antibiotics and chemotherapy on the microbiome and its antitumor effects.

### Microbiome as a potential mediator of therapy success

The impact of drugs like 5-FU or cephalosporins on the microbiome is substantial and often underappreciated, exerting significant influence on infections, tumor therapy, and the manifestation of (toxic) side effects (Dubin et al., 2016; Hobson et al., 2022). Compelling evidence suggests a strong correlation between the intestinal microbiota and the response to immunomodulatory therapy like chemotherapy, enhancing or reducing (chemo-) therapy success. Moreover, the composition of the baseline microbiota profile appeared to be linked to the occurrence of infectious complications resulting from chemotherapy. Notably, chemotherapy and immunotherapy induced significant alterations in the gut microbiota composition, potentially impacting treatment effectiveness (Aarnoutse et al., 2019; Montassier et al., 2016). Furthermore, the Capecitabin/Oxaliplatin chemotherapy regimen has been shown to induce substantial alterations in the intestinal microbiota. Chemotherapy, in general, can lead to significant changes in the gut microbiota, including an increase in pathogenic and treatment-adapted bacteria, as well as a decline in beneficial microbes, which can potentially disrupt intestinal homeostasis. Interventions targeting the microbiota potentially benefit patients in the postoperative phase of clinical management (Kong et al., 2019). Additionally, antibiotics have been demonstrated to modulate the microbiome and reduce tumor development in experimental models, suggesting a potential modulatory effect on cancer progression (Dapito et al., 2012; Yoshimoto et al., 2013; Ferreri et al., 2012; Lecuit et al., 2004; Senff et al., 2008; Dove et al., 1997; Reddy et al., 1974; Reddy et al., 1975; Vannucci et al., 2008; Lucas et al., 2017; Philpott et al., 2014).

### Trade-off

In this study, it was suspected that the delay in the subsequent chemotherapy cycle due to infections and antibiotics therapy (Figure 1), aimed at mitigating complications, would likely result in poorer staging outcomes due to the cumulative loss of antitumor therapy over time. This highlights a therapeutic trade-off between maximizing anticancer efficacy and minimizing toxicity to preserve quality of life. Reducing treatment intensity may improve tolerability but can compromise dose intensity and oncological outcomes, making the definition of an optimal therapeutic window crucial (Benasso et al., 1995). However, the clear superiority of the CAB group over the reference group in terms of disease stability, tumor regression, and less tumor progression suggests the potential antitumor effect of the antibiotics. It is also possible that CAB, like other antibiotics (Jetté et al., 2008), influences 5-FU sensitivity and response, contributing to the observed effects.

### Adjustments in metastatic diseases

Regarding the need for chemotherapy adjustments, CAB patients and especially patients with metastatic tumors required modifications of their chemotherapy regimens. This is unsurprising, as research highlights the complex interplay between oncogenes and cellular signaling networks in cancer progression (Klampfer et al., 2005), such as metastases, known to promote further DNA mutations, leading to unfavorable responses to chemotherapy and ultimately reducing life expectancy (Douillard et al., 2013). Furthermore, developing resistance to 5-FU-containing chemotherapies is a well-documented and serious issue, especially in advanced metastatic cancer, where cells adapt and build resistance strategies (Sethy and Kundu, 2021). CAB, similar to many antibiotics, may interact with other chemotherapeutic agents, necessitating adjustments in treatment (Jetté et al., 2008). However, due to the largely successful antimicrobial therapy, side effects (Table 4), and staging comparison (Figure 2), there is no indication that the co-administration of CAB with the investigated chemotherapeutic agents interfered with treatment efficacy or adversely impacted clinical outcomes. The reasons for altered therapy responses in metastatic patients are varied and complex. Factors such as the heavy burden of infectious disease, compounded by advanced diagnoses, limited quality of life, and reduced tolerance to doses and side effects, are possible explanations. This area presents opportunities for further research with specific questions and larger quantitative studies.

### Side effects and confounding factors

Regarding side effects, the present study demonstrates that in the CAB group, infection- and antibiotic-associated adverse events were more frequent, likely due to the intensive use of antimicrobial agents and the severity of infections. Nevertheless, when comparing the overall toxicity profiles, the cumulative burden of adverse events in the CAB group did not exceed that of the reference group, where toxicity was predominantly related to chemotherapeutic agents such as 5-FU, Capecitabine, Trifluridine/Tipiracil, and other drugs used in the regimens. For instance, despite infection-associated bad physical condition in cancer patients, the incidence of severe side effects in CAB-treated patients is not greater than that observed in the reference group. Side effects affecting patients’ wellbeing, such as pain, are also related to infection and clinical stay. For instance, infections, especially in immunocompromised patients, often lead to inflammatory responses, which can cause pain in various tissues or organs (Mohammed et al., 2014), explaining the increased analgesic requirements in the CAB group. Furthermore, an oxaliplatin-containing chemotherapy regimen may also be a factor leading to side effects. Since 50% or more of the patients with chemo regimen adjustments were treated with oxaliplatin containing regimens, it is reasonable to hypothesize that oxaliplatin leads to increased side effects and drug interactions, possibly due to the platinum content. For instance, studies have demonstrated that oxaliplatin or platinum-based chemotherapy regimens are linked to elevated incidence of toxic effects, notably in the hematological, gastrointestinal, and neurological domains. Platinum-based drugs like oxaliplatin are recognized for their elevated systemic toxicity, resulting in limited clinical applicability due to the development of drug resistance (Cassidy and Misset, 2002; Zhang et al., 2022). Oxaliplatin-containing chemotherapy may result in overrepresenting side effects not caused by CAB, 5-FU, Capecitabine, or Trifluridine/Tipiracil. The extensive medication plans and the dynamic course of the disease make it challenging to interpret the underlying reasons for these results in the patient population. In such cases, it is difficult to determine whether DDIs or the disease is the primary cause (Neuberger, 2021).

### CAB application strategy

While conventional antimicrobial strategies often rely on the concurrent use of multiple agents to reduce toxic doses and achieve broad-spectrum coverage, the findings suggest that a sustained, targeted CAB monotherapy may offer a more favorable balance between efficacy and tolerability. Interestingly, the findings support the hypothesis that prolonged and exclusive (sole short-term) administration of CAB may be associated with a more favorable side effect profile compared to broader antibiotic combination regimens (Figure 4). These observations underline the potential of optimized, agent-specific antibiotic strategies to minimize treatment-related toxicity without compromising therapeutic effectiveness.

This, in turn, enhances patients’ quality of life and their capability to resume other therapies. There are numerous potential reasons for the particularly positive response to CAB, primarily when used as a standalone antibiotic.

### Enzymatic activity in microbiota

Side effects resulting from chemotherapy may be associated with bacterial dysbiosis, which can be mitigated through the preventive use of antibiotics (Ting et al., 2022). On a biological level, the enzymatic activity of bacterial β-glucuronidase of the microbiome significantly contributes to the metabolism of xenobiotics, thereby influencing the efficacy and adverse effects of specific antitumor medications. However, antibiotics or inhibitors targeting bacterial β-glucuronidase can effectively prevent these unwanted effects. Consequently, there is a potential avenue for designing drugs that can inhibit detrimental enzyme activities in crucial microbial symbiotes, thereby augmenting the effectiveness of chemotherapy (Haiser and Turnbaugh, 2012; Wallace et al., 2010).

### Blood count variability

Finally, only subtle variations were observed when comparing blood counts between the CAB and reference groups (data not shown), including isolated cases of thrombocytopenia, neutropenia, and leukocytosis. Leukocytosis occurred at comparable rates in both groups, whereas thrombocytopenia was observed exclusively in the CAB group. Although neutropenia appeared to occur less frequently in the CAB group than in the reference group (Table 4), it remains uncertain whether this reflects a real reduction, given the limitations of the dataset. It is worth noting that alterations in blood count are inherently difficult to interpret and may be confounded by the effects of the underlying infection itself and by treatment-related supportive therapies, such as G-CSF (i.e., filgrastim, pegfilgrastim). Moreover, blood counts declined progressively in some patients over successive chemotherapy cycles, making definitive analysis challenging. Thus, future studies focusing on hematologic evaluations are warranted to accurately assess the potential effects of CAB on hematologic parameters.

## Limitation

The authors acknowledge that the study’s small sample size may reduce statistical power and limit the generalizability of the findings. Additional limitations include the concurrent administration of other chemotherapeutic agents within treatment regimens and the use of supplementary antibiotics to expand antimicrobial coverage in infectious situations. This study shows that compared to the reference group, CAB patients took a median of 6 days longer to start their next chemotherapy cycle. However, it is worth mentioning that the possibility that additional chemotherapy drugs in the regimen might negatively impact the results cannot be excluded. Nevertheless, CAB therapy provides a highly favorable prognosis for cancer development without an increased side effect perceptibility. This finding was observed but not further investigated. The main reason was the limited patient pool size, which hindered the identification of a more precise reference group that would also determine the exact therapies used for the patients. Nevertheless, the findings are intended to lay the groundwork for future research on a larger scale to address the limitations encountered in this study. The ultimate aim is to contribute to the development of evidence-based strategies to improve the management of cancer patients with concomitant infections.

## Conclusion

DDIs between antibiotics and anticancer drugs are a significant concern that warrants further research, particularly in high-risk patients, such as cancer patients, for developing personalized treatments that improve outcomes and address the challenge of multi-resistant pathogens (Radu et al., 2024). This study demonstrates a significant positive impact of CAB therapy on cancer patients, particularly in terms of tumor development, where a decrease in the extent of tumor progression and more stable disease/tumor regression were observed in the CAB group compared to the reference group. Moreover, side effects were fewer or similar in the CAB group compared to the reference group, without having the heavy burden of infection, but with a notable reduction in chemotherapy-associated side effects in the CAB group.

Studies on the sole and more extensive use of CAB may indicate a consistent trend toward positive outcomes, suggesting a potential therapeutic advantage that warrants further investigation. Although a 6-day delay in chemotherapy application occurred, predominantly positive outcomes regarding anticancer and anti-infection effects were observed. Despite the small patient pool, significant effects were achieved. The impact of metastasis and advanced cancer on the outcomes, concerning the even smaller patient number, remains unclear and warrants further investigation. Additionally, the influence of infection diagnosis on anticancer effects and the specific CAB used, which may differ slightly from those in the reported laboratory-clinical studies, require further exploration. There remains significant potential for future research within an appropriate framework. In summary, it is essential to note that using CAB replaces the traditional side effects of chemotherapy with the known side effects of antibiotics, without increasing the quantity of side effects. This positive effect was observed despite the potential for additional DDIs arising from the combined chemotherapy treatment regimen and the multi-drug infection management. The findings present significant promises for treating infectious intestinal cancer and underscore the crucial need to comprehend DDIs thoroughly in this context. Despite existing reports highlighting DDIs involving antibiotics and other drug classes (Pai et al.), a more profound and clinically grounded understanding is still needed to evaluate in a large-scale study DDIs between antibiotics and anticancer agents.

## Data Availability

The original contributions presented in the study are included in the article/Supplementary Material, further inquiries can be directed to the corresponding author.
