# Correction: Drug interactions between cephalosporins and 5-FU-based chemotherapy in the treatment of patients with gastrointestinal cancer - an exploratory cohort analysis

**DOI:** 10.3389/fphar.2025.1721652

**Published:** 2025-10-20

**Authors:** Robert Siegel, Sophie Schlosser-Hupf, Martina Müller-Schilling, Samer A. Kharroubi, André Gessner, Nahed El-Najjar

**Affiliations:** ^1^ Institute of Clinical Microbiology and Hygiene, University Hospital Regensburg, Regensburg, Germany; ^2^ Department of Internal Medicine I, Gastroenterology, Hepatology, Endocrinology, Rheumatology, Immunology, and Infectious Diseases, University Hospital Regensburg, Regensburg, Germany; ^3^ Department of Nutrition and Food Sciences, Faculty of Agricultural and Food Sciences, American University of Beirut, Beirut, Lebanon; ^4^ Department of Pharmacology and Toxicology, Faculty of Medicine, American University of Beirut, Beirut, Lebanon

**Keywords:** drug-drug interactions, cephalosporins, 5-fluorouracil, sepsis, oncology, adverse drug reactions

Affiliation “Institute of Clinical Microbiology and Hygiene, University Hospital Regensburg, Regensburg, Germany” was erroneously omitted for author **Nahed El-Najjar**.

The original article has been updated.

