# Supplementary material for: Drug interactions between cephalosporins and 5-FU-based chemotherapy in the treatment of patients with gastrointestinal cancer - an exploratory cohort analysis
Source: Front Pharmacol. 2025 Oct 7;16:1652957. doi: 10.3389/fphar.2025.1652957 (PMC12580600; doi:10.3389/fphar.2025.1652957)
Supplement: Supplementary file 1 [file DataSheet1.zip › Summary of the Codes used in the SPSS file.docx]

Table 1:

Age

**CROSSTABS
  /TABLES=age BY Assignment
  /FORMAT=AVALUE TABLES
  /STATISTICS=CHISQ
  /CELLS=COUNT
  /COUNT ROUND CELL.**

Sex

**CROSSTABS
  /TABLES=Sex BY Assignment
  /FORMAT=AVALUE TABLES
  /STATISTICS=CHISQ
  /CELLS=COUNT
  /COUNT ROUND CELL.**

Tumor location

**CROSSTABS
   /TABLES=TumorLocation BY Assignment
  /FORMAT=AVALUE TABLES
  /STATISTICS=CHISQ
  /CELLS=COUNT
   /COUNT ROUND CELL.**

Cancer extent

**CROSSTABS
  /TABLES=CancerExtent BY Assignment
  /FORMAT=AVALUE TABLES
  /STATISTICS=CHISQ
  /CELLS=COUNT
  /COUNT ROUND CELL.**

Antibiotics used in the last 3 months

**CROSSTABS
   /TABLES=AntibioticsLast3Mo BY Assignment
   /FORMAT=AVALUE TABLES
   /STATISTICS=CHISQ
   /CELLS=COUNT
   /COUNT ROUND CELL.**

Comorbidities

**CROSSTABS
   /TABLES=Comorbidities BY Assignment
   /FORMAT=AVALUE TABLES
   /STATISTICS=CHISQ
   /CELLS=COUNT
   /COUNT ROUND CELL.**

Table 2:

Additional time to rest

**T-TEST GROUPS=Assignment(0 1)
   /MISSING=ANALYSIS
   /VARIABLES=AdditionalRest
   /ES DISPLAY(TRUE)
   /CRITERIA=CI(.95).**

Table 3:

Staging results

**CROSSTABS
   /TABLES=Staging BY Assignment
   /FORMAT=AVALUE TABLES
   /STATISTICS=CHISQ
   /CELLS=COUNT
   /COUNT ROUND CELL.**

Table 4:

Side effects on CAB duration

**T-TEST GROUPS=SideEffects(0 1)
   /MISSING=ANALYSIS
   /VARIABLES=CABduration
   /ES DISPLAY(TRUE)
   /CRITERIA=CI(.95).**

Side effect comparison CAB & Ref

**CROSSTABS
   /TABLES=SideEffectsCABandREF BY Assignment
   /FORMAT=AVALUE TABLES
   /STATISTICS=CHISQ
   /CELLS=COUNT
   /COUNT ROUND CELL.**
